# Improvement of experimental testing and network training conditions with genome-wide microarrays for more accurate predictions of drug gene targets

**DOI:** 10.1186/1752-0509-8-7

**Published:** 2014-01-20

**Authors:** Lisa M Christadore, Lisa Pham, Eric D Kolaczyk, Scott E Schaus

**Affiliations:** 1 Department of Chemistry, Boston University, Boston, MA, USA; 2 Program in Bioinformatics and Mathematics, Boston University, Boston, MA, USA; 3 Department of Mathematics and Statistics, Boston University, Boston, MA, USA

## Abstract

**Background:**

Genome-wide microarrays have been useful for predicting chemical-genetic interactions at the gene level. However, interpreting genome-wide microarray results can be overwhelming due to the vast output of gene expression data combined with off-target transcriptional responses many times induced by a drug treatment. This study demonstrates how experimental and computational methods can interact with each other, to arrive at more accurate predictions of drug-induced perturbations. We present a two-stage strategy that links microarray experimental testing and network training conditions to predict gene perturbations for a drug with a known mechanism of action in a well-studied organism.

**Results:**

*S. cerevisiae* cells were treated with the antifungal, fluconazole, and expression profiling was conducted under different biological conditions using Affymetrix genome-wide microarrays. Transcripts were filtered with a formal network-based method, sparse simultaneous equation models and Lasso regression (SSEM-Lasso), under different network training conditions. Gene expression results were evaluated using both gene set and single gene target analyses, and the drug’s transcriptional effects were narrowed first by pathway and then by individual genes. Variables included: (i) Testing conditions – exposure time and concentration and (ii) Network training conditions – training compendium modifications. Two analyses of SSEM-Lasso output – gene set and single gene – were conducted to gain a better understanding of how SSEM-Lasso predicts perturbation targets.

**Conclusions:**

This study demonstrates that genome-wide microarrays can be optimized using a two-stage strategy for a more in-depth understanding of how a cell manifests biological reactions to a drug treatment at the transcription level. Additionally, a more detailed understanding of how the statistical model, SSEM-Lasso, propagates perturbations through a network of gene regulatory interactions is achieved.

## Background

RNA microarrays have had a major impact on both experimental and computational biology. They have played a role in predicting molecular targets and bioactive compound modes-of-action [1-3], they have helped identify genes responsible for disease- and environmental-induced phenotypes [4-6]. At the same time, statistical methods for interpreting genome-wide microarray data have progressed over the past decade. Drug target identification methods have gone from labor-intensive techniques, like chemogenomic fitness or haploinsufficiency profiling [7-11], to more efficient, statistically driven models such as those based on network-filtering [12-16] and network topology association [17]. 

Supervised learning methods like support vector machines have also been widely used to develop statistical methods that predict drug-protein interactions [18-22]. These methods employ training networks, constructed from protein-ligand binding data, known protein sequences, compound similarity scores, and in the case of Campillos et al., known drug side effects. Similar to our method, these training networks capture interaction “patterns” between two molecules (eg, ligand-protein) to predict known and new drug targets. Although unlike our method, these patterns are typically taken as known input, whereas in SSEM-Lasso, they are learned from the microarray data.

Accurate interpretation of transcriptional changes resulting from genome-wide microarray data can be influenced by different variables, including those manifested by the experimental biologist and the computational biologist. These variables are especially critical for drug treatment studies, because drugs tend to produce multi-gene and/or off-target perturbations [14,23,24]. For example experimental variables, such as RNA quality, microarray preparation, nutrients, genetic background, and duration and strength of drug treatment can all play a role in the final gene target analysis [25-27]. Similarly, information incorporated into any training, or learning, phase that is used to infer a gene interaction network or similar model structure, can impact results at the gene level. The potential effects of both biological and computational conditions – separately, or on their own – are widely acknowledged. Nevertheless, it appears there is little work explicitly examining how these two types of conditions *interact with each other* to produce accurate and reliable molecular target predictions.

**Figure 1:**
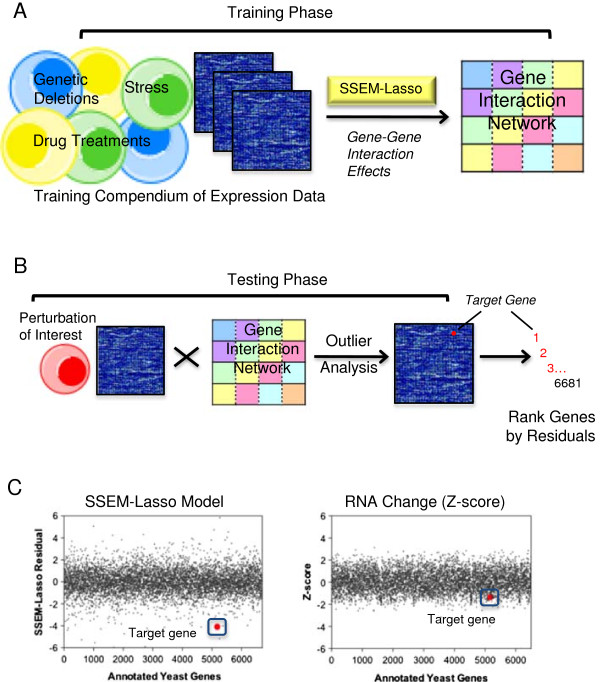
**SSEM-Lasso network-inference methodology for prediction of gene targets. (A)** In the training phase, transcript signals derived from a training compendium of Affymetrix yeast expression data estimated a gene interaction network using sparse simultaneous equation models and Lasso regression (SSEM-Lasso). The gene interaction network accounted for every gene’s effect on another gene within the compendium and was used to infer subsequent experimental perturbations of interest. **(B)** In the testing phase, experimental expression data was processed with the gene interaction network, and mRNA transcript signals were adjusted based on all inferred gene regulatory effects in the network. An outlier analysis yielded residual values for every gene in the compendium. Residuals were ranked by their absolute values, and genes with lower ranks were considered more accurate predictions of directly targeted genes of the experimental perturbation. **(C)** SSEM-Lasso “resolves” experimentally perturbed genes out of the background gene-gene interaction “noise” in the network. This results in a more stringent gene-target filter in comparison to standard z-score computation. The data shown is from a *top2Δ/TOP2* heterozygous yeast deletion microarray experiment conducted in-house. The gene target, *TOP2*, is significantly perturbed when evaluated with SSEM-Lasso compared to the RNA z-score prediction.

SSEM-Lasso uses a network-based approach consisting of two phases, training and testing. In the training phase, the method learns a collection of gene-gene interaction effects from compendium of microarray experiments (training compendium), which are captured in an interaction network (Figure 1A). Then, in the testing phase, the method identifies genes experiencing an additive shift in their mean transcript levels in response to an external perturbation (eg, drug treatment), after adjusting for the inferred gene-gene interactions (Figure 1B). Transcript residuals resulting from this step are ranked by their absolute values for all annotated genes in the compendium (Figure 1B). Genes with low ranks (large residuals) are genes SSEM-Lasso distinguishes as standing out from the background gene regulatory effects. They are flagged as potential targets of the external perturbation of interest. As a result, SSEM-Lasso has the ability to significantly narrow the gene target window in comparison to RNA change z-score computations (Figure 1C).

SSEM-Lasso is unlike standard machine learning methods in that it is based on an explicit model of how perturbations propagate through a network of gene regulatory effects, in the form of a system of sparse simultaneous equations (which, in turn, may be viewed as a stochastic version of standard first-order differential equations) [12]. Other machine learning approaches typically have been based on models defined through relationships learned between known drug-target interactions and various biological inputs [18-22]. SSEM-Lasso also differs from machine learning in the data being analyzed and the interaction pairs being identified. SSEM-Lasso uses microarray gene expression data from hundreds of experiments carried out under varying experimental conditions. The machine learning methods discussed above use for example, protein-ligand interaction and compound 2-D similarity data listed in conventional databases like KEGG [28], BRENDA [29], and SIMCOMP [30].

SSEM-Lasso’s mathematical properties have been fully characterized [12]. The method has achieved improved sensitivity and accuracy over the RNA z-test method (ie, ranking genes as targets based on normalized expression data, without any additional modelling or processing) and a competitive, alternative network-inference method, MNI [14]. Furthermore, the two phases of SSEM-Lasso allow for modifications of both experimental variables (testing phase) and computational variables (training phase) to optimize drug target predictions.

SSEM-Lasso is applicable to a range of perturbation experiments. The method does not impose explicit constraints on the experimental conditions, such as particular deletion strain, inducible expression system, or time-course conditions, for inferring gene network interactions. SSEM-Lasso has been shown to perform well in the context of *in silico* experiments. Furthermore, it has proven successful at predicting gene targets of *S. cerevisiae* haploid and diploid deletion strains. However, the method requires further optimization for predicting drug gene targets (Additional file 1). This is an anticipated caveat due to the biologically complex nature of a chemical perturbation. Given this combination of features, we determine SSEM-Lasso ideal for our goal of investigating experimental-computational dynamics, and at the same time, we seek to improve its performance with drug gene target predictions.

**Figure 2:**
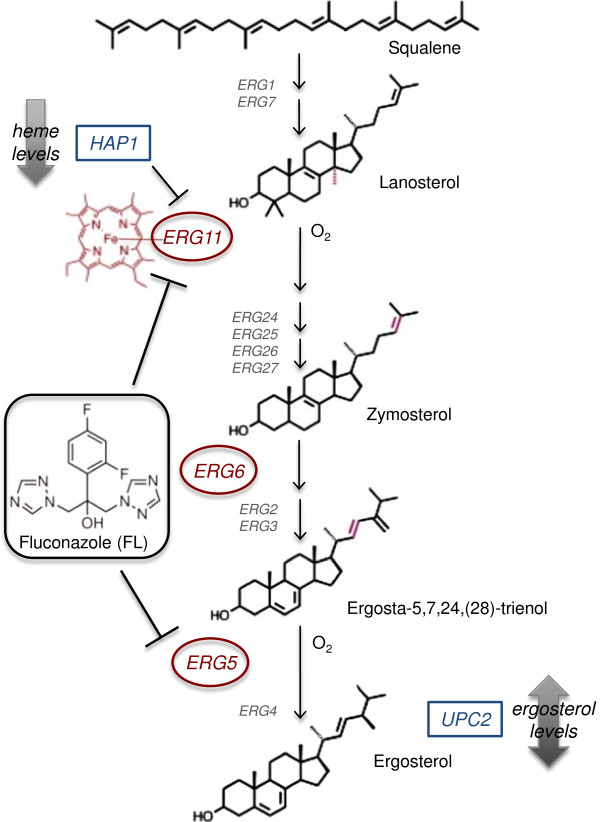
**Summary of FL enzymatic and transcription factor gene targets.** Genes affected by fluconazole (FL) investigated in this study are enzymes along the ergosterol biosynthetic pathway (circles) and transcription factors directly regulated by sterol and heme levels (squares). *ERG11*, the gene that codes for lanosterol C-14-α demethylase, is the primary target of FL. CYP450 C-22 sterol desaturase, *ERG5* (circle), is also a target of FL and its enzymatic activity is inhibited upon FL binding. FL’s nitrogen interacts with the heme groups of both Erg11p and Erg5p disrupting normal ergosterol synthesis and affecting downstream enzymatic reactions, including those performed by Δ[24]-sterol C-methyltransferase, Erg6p (circle). FL disruption of sterol biosynthesis additionally affects *UPC2* (square), the gene that encodes for a sterol regulatory binding protein responsible for increased transcription of ERG genes upon sterol depletion. FL induces defective respiration due to its disruption of heme and oxygen levels. Therefore, *HAP1* (square), a transcription factor responsible for regulating *ERG11* expression under hypoxic conditions, is also targeted.

We use the model organism, *S. cerevisiae*, and a compound with a known mechanism of action, fluconazole (FL), to explore how choice of variables in testing and training phases influences the quality of final gene target predictions. FL specifically binds and inhibits cytochrome P450 (CYP450)-dependent lanosterol C-14-α demethylase (Erg11p) [31,32], an essential hemoprotein in the ergosterol biosynthesis pathway (Figure 2). Ergosterol is the principle component of yeast cell membranes, similar to cholesterol in animal cells. FL inhibition of Erg11p causes the accumulation of toxic 14-α-methylated sterols and ergosterol depletion [33,34]. This results in increased cell membrane permeability and asymmetry and irregular sphingolipid, phospholipid and long-chain fatty acid synthesis [35-37]. Additionally, Erg11p is dependent on oxygen and heme production, and *ERG11* deletion strains are nonviable under aerobic growth conditions. Therefore, Erg11p disruption by FL can also lead to defects in heme biosynthesis and mitochondrial respiration [38-40].

In addition to *ERG11*, *ERG6*, *UPC2* and *HAP1* are impacted by sterol and heme depletion. *ERG6* encodes for Δ(24)-sterol C-methyltransferase, an enzyme that functions downstream of Erg11p in ergosterol biosynthesis and is responsible for the transmethylation of zymosterol to fecosterol [36] (Figure 2). Although Erg6p is not the direct gene target of FL, nor is it essential for cell growth, Erg6p regulates appropriate sterol and fatty acid composition and distribution, and is therefore required for normal cell membrane permeability and rigidity [41-43].

Sterol regulatory element binding protein, Upc2p, is a member of the Zn_2_-Cys_6_ transcription factor family required for exogenous sterol uptake during anaerobic growth. Upc2p, with Ecm22p, regulates the transcription of late stage ergosterol biosynthesis genes in response to azole-induced sterol depletion [44,45] (Figure 2). Its DNA binding sequence is conserved between *C. albicans* and *S. cerevisiae* and is in a region of the *ERG11* promoter critical for azole induction of *ERG11* expression [46,47].

Heme-activator protein, Hap1p, is a transcription factor that controls the expression of aerobic and anaerobic genes through both its interactions with heme and transcriptional control of heme-dependent repressor of hypoxic genes, [48,49]. Hap1p binds the promoters of *ERG5* and *ERG11* under both aerobic and hypoxic conditions, however it is most active in repressing these genes’ expressions under hypoxic conditions [48,50] (Figure 2). *ERG11* expression through induction of *ROX1* is also regulated by Hap1p. Additionally, Hap1p controls Upc2p expression to maintain basal expression levels of *ERG* genes. However upon sterol depletion, Upc2p no longer requires Hap1p to transcribe *ERG* genes [51]. These genes – *ERG6*, *UPC2*, and *HAP1* – are therefore interconnected and exemplary targets to further investigate FL-induced expression changes.

The expression of the CYP450 C-22 sterol desaturase, *ERG5*, is indirectly investigated in this study through the haploid heme deletion strain, *hem1Δ*. The *hem1Δ* strain cannot synthesize δ-aminolevulinic acid, a precursor in heme synthesis. In the absence of heme, Hap1p binds the *ERG5* promoter to suppress expression of *ERG5*. Therefore *hem1Δ* mutants contain little to no Erg5p mRNAs [48]. Additionally, Erg5p can be directly bound and inhibited by azoles, like FL (Figure 2) [52]. Thus, *ERG5* is considered the primary target of the *hem1Δ* strain and an additional FL-targeted gene in this study.

This study contributes to the larger goal of improving microarrays for therapeutic discovery and development. Using a model organism and an established drug with known target genes and pathways, we are able to identify conditions that both the experimentalist and computational biologists can fine-tune to more accurately predict drug effects at the gene expression level. Herein we present a step-wise strategy through SSEM-Lasso that scientists can take to optimize drug gene target predictions at the genome-wide level.

## Results

### Testing phase (experimental) variations for predicting FL gene targets

The overall goal of the testing phase was to determine if changes in experimental variables, or “input”, altered SSEM-Lasso ranks, or “output”, of drug gene targets (Figure 1B). We assessed how well SSEM-Lasso identified a target gene relative to off -target, or orthogonal, genes over changing experimental conditions. This was evaluated by comparing the rank of the target gene relative to the rank of off-target genes.

**Figure 3:**
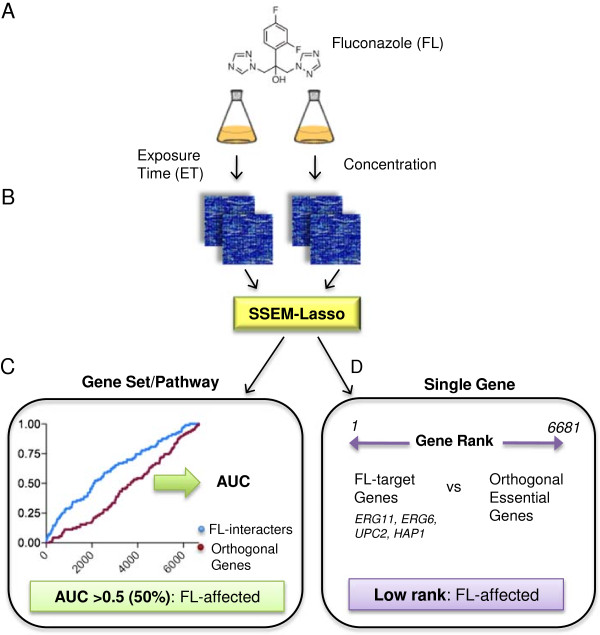
**Experimental methodology for fluconazole treatment experiments. (A)** Wild-type yeast cells (BY4741) were treated with fluconazole (FL) at various exposure times and concentrations under constant growth conditions. **(B)** RNA purification, amplification and hybridization to Affymetrix YG S98 GeneChips were carried out and raw signal data was RMA-normalized and processed with SSEM-Lasso to determine residuals and subsequent ranks for all genes in the network. Two replicates for each condition were performed from two separate FL treatment experiments. **(C)** Gene set analysis detected gene perturbations of multiple, related genes across an increasing SSEM-Lasso rank threshold, resulting in a sensitivity vs. rank threshold curve (ROC curve) for each experimental condition. Area under each ROC curve was calculated, averaged for each duplicate experiment and reported as AUC%. AUC% values >0.5 (50%) indicated greater FL perturbation on the gene set. Gene set analyses were conducted for target pathway, FL-interacters (blue), and orthogonal pathways (purple). **(D)** Single gene analysis predicted FL perturbation on gene targets, *ERG11*, *ERG6*, *UPC2* and *HAP1*, for every FL treatment condition. Target gene ranks were compared to the average ranks of six orthogonal genes. Low ranked genes were considered more accurately perturbed by FL. Ranks were averaged for two replicate experiments.

Studies have shown that desired physiological responses can be achieved by controlling the duration a drug is in contact with the host organism and the concentration of drug administered. This in turn can lead to more effective treatment strategies [53,54]. In the testing phase we regulated these critical treatment variables, exposure time and concentration, while maintaining constant the training compendium used to infer the gene network interactions. Figure 3 outlines the experimental workflow. Wild-type *S. cerevisiae* cells were treated with FL and harvested at either varying exposure times (ET) or concentrations in aerobic, batch culture conditions (Figure 3A). Affymetrix microarray experiments were carried out in duplicate, and transcript signals were RMA-normalized and processed with SSEM-Lasso (Figure 3B). For all testing phase variations, the original training compendium from Cosgrove et al. was used to infer the gene interaction network.

Two analyses of SSEM-Lasso output – gene set and single gene – were conducted. For the gene set analysis, genes representative of 6 different biological pathways were grouped to form 1 target and 5 orthogonal gene sets. The target gene set unique to FL action, called “FL-interacters”, was comprised of genes affected by FL and/or associated with *ERG11*. FL-interacters included genes from ergosterol, terpenoid backbone, fatty acid and sphingolipid biosynthesis pathways, and sterol transport, heme and oxygen sensing pathways (Additional file 2). The five orthogonal gene sets included genes from: DNA replication and repair, pyrimidine biosynthesis and metabolism, RNA transport, glycolysis and pentose phosphate and mitosis (Additional files 3, 4, 5, 6, 7).

Gene set analysis was carried out as follows: Each gene within a gene set was detected across an increasing gene rank threshold, resulting in a receiver operating characteristic, or ROC, curve for an FL treatment experiment. ROC curves are graphical plots of the true positive rate against the false positive rate as the discrimination threshold is varied. Areas under ROC curves were converted into percentages (AUC%) and compared across FL treatments (Figure 3C). Steeper ROC curves result in larger AUC percentages. The expected AUC from random guessing is 0.5, or 50%. AUC values falling below 0.5 denoted a higher false positive rate than random guessing, while AUC values above 0.5 denoted more true positives than what were expected with random guessing. We considered treatment experiments with AUCs >0.5 to experience stronger biological pathway effects than experiments with AUC%s ≤0.5.

For single gene analysis, FL perturbation of its primary target, *ERG11*, was tracked along with FL targets associated with ergosterol biosynthesis, sterol transcription initiation and heme and oxygen transcriptional regulation, *ERG6*, *UPC2* and *HAP1*, respectively (Figure 3D). Ranks for orthogonal genes essential to cellular survival, *MPS1*, *ADE13*, *TOP2*, *CDC9*, *PAB1* and *UBA1*, were also monitored as indicators of FL off-target effects. Lower ranks suggested more accurate prediction of FL action on an individual gene (Figure 3D). Rank percentiles were also computed to assess how well a gene ranked relative to the background set of all genes.

### Training phase (network inference) variations for improving FL single gene target predictions

In addition to the testing phase strategy, modifications to the gene interaction network in the training phase were conducted. The overall goal of the training phase was to determine if changes to the network increased or decreased ranks of gene targets.

**Figure 4:**
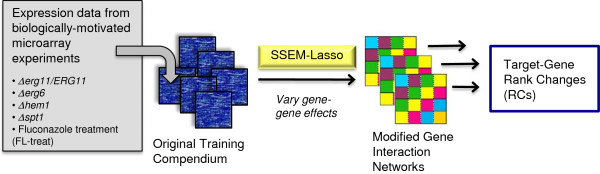
**Network training methodology for fluconazole treatment experiments. ***S. cerevisiae* expression data from 5 microarray experiments were individually added to the original training compendium from Cosgrove et al. Separate SSEM-Lasso runs were performed on each of the modified training compendiums resulting in unique changes to the gene interaction network. Subsequent changes to gene ranks were reported, along with percentile values to evaluate how much “better” or “worse” a gene ranked with a given, modified training compendium.

Variations to the training phase involved the addition of biologically-motivated microarray expression data into the original training compendium from Cosgrove et al. Subsequent modifications to gene-gene interaction “patterns” propagated through the network resulted in changes of varying degrees in SSEM-Lasso rank predictions (Figure 4). Two measurements, rank change (RC) and RC percentile, reflected how strongly network variations affected SSEM-Lasso predictions. If RC of a target gene was positive, prediction of the gene perturbation was said to “improve”. RC of a target gene was compared against all gene RCs within an experiment by calculating a percentile of RC, or the percentage of genes with a RC as high or higher than the target gene. The RC percentile provide a quantitative assessment of RC. Thus, a tuneable network inference variable was established in the training phase.

### Testing phase: Exposure time variations have stronger effects on FL gene set than on FL single gene targets

The first experimental condition manipulated in the testing phase was FL exposure time (ET). The length of FL treatment in a population of unsynchronized cells was varied between one and four ETs, at constant FL concentration (GI_10_). For each ET experiment, results from both gene set (AUC%) and single gene (rank and percentile) were reported.

**Figure 5:**
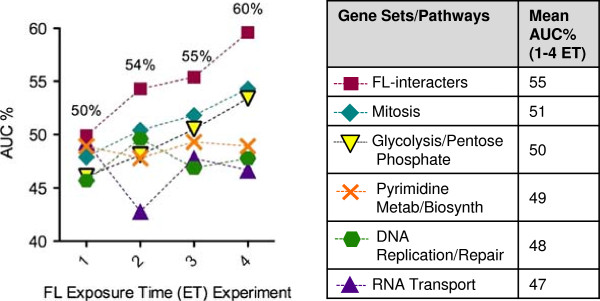
**Exposure time effects on gene set (AUC%) analysis.** Areas under each sensitivity vs. rank threshold curve (ROC curve) for FL-interacters and orthogonal gene sets/pathways were converted to percentages (AUC%s) and plotted for each FL ET experiment. Mean AUC%s (ET 1 to 4) for each gene set were computed and compared in the table. Larger AUC% values indicated better prediction of FL action on a gene set. AUC% values were the averages of two replicates.

Gene set analysis for FL-interacting genes resulted in individual AUC%s for each ET experiment, which were plotted for each of the 6 gene sets as shown in Figure 5. Longer FL ETs correlated with higher AUC%s. From one to two ETs, AUC% increased noticeably from 50 to 55%, and at four ETs AUC% was at its highest of 60% (Figure 5, squares). These results indicated that incubation of cells with FL for four ETs was the optimal time point for SSEM-Lasso to predict FL effects on multiple, target genes.

AUC%s for the orthogonal gene sets were also plotted, and similar improvement trends were observed for mitosis and glycolysis/pentose phosphate gene sets (Figure 5, diamonds and inverted triangles). SSEM-Lasso likely identified similar trends for these pathways, because they are also influenced by FL treatment. Severe ergosterol depletion interferes with sparking functions of ergosterol [55]. Hence, genes involved in mitosis that are critical to late stages of the cell cycle may have been dysregulated upon FL treatment. Additionally, FL interrupts heme function, and thus the cell population may have adapted to increased hypoxic conditions by shifting from glucose metabolism via respiration to anaerobic fermentation [56], thus affecting genes involved in glycolysis/pentose phosphate. Yet, the other orthogonal pathways, RNA transport, pyrimidine biosynthesis/metabolism, and DNA replication/repair, did not reproduce the same improvement trend nor exhibit AUC% values >50% (Figure 5). Overall, the average AUC%s for each of the 5 orthogonal gene sets were lower than those for FL-interacter gene set, demonstrating that FL exerted its strongest effects on the FL-targeted gene set (Figure 5).

**Figure 6:**
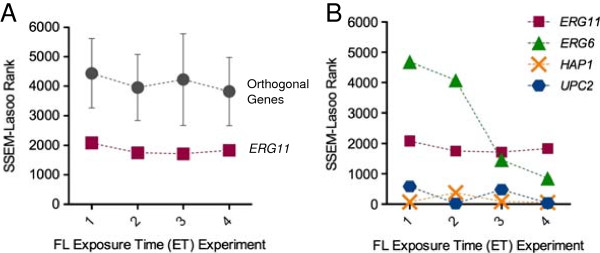
**Exposure time effects on single gene (rank) analysis. (A)** SSEM-Lasso ranks of FL’s primary gene target, *ERG11* (squares), were compared to gene rank averages for six orthogonal genes, *MPS1*, *ADE13*, *TOP2*, *CDC9*, *PAB1* and *UBA1* (circles), across increasing ETs. Error bars represent standard deviation for orthogonal gene ranks. **(B)** SSEM-Lasso ranks of all FL targets, *ERG11* (squares), *ERG6* (triangles), *UPC2* (hexagons) and *HAP1* (crosses) versus FL ET experiments. Cells were treated with FL concentrations that corresponded to increasing growth inhibitory percentages, GI%s (x-axis). Lower ranks indicated better prediction of FL action on an individual gene. All ranks were the averages of two replicates.

Single gene targets also were tracked across ETs. *ERG11* ranked consistently lower than the collective population of orthogonal genes (Figure 6A), suggesting SSEM-Lasso’s predictions were more specific for FL action on its therapeutic target.

Interestingly, changing ET (1 ➔ 4) did not improve the algorithm’s predictions*,* and *ERG11* rank remained constant, between 1716 and 2080 (Figure 6A, squares), or in the 70^th^ percentile of ranks (Table 1). This was unlike the FL-interacter gene set results, which demonstrated a distinct trend with increasing ET (Figure 5).*ERG6* was the only FL target that followed a distinct trend, in which *ERG6* rank decreased at higher ET. At three and four ETs, *ERG6* ranked in the 78th and 87th percentiles respectively, which were lower than *ERG11* ranks (Figure 6B, triangles, Table 1). Ranks for *UPC2* and *HAP1* were consistently lower than both *ERG11* and *ERG6. UPC2* and *HAP1* ranked in the 93^rd^ to 99.7^th^ percentiles for two, three, and four ETs. Similar to *ERG11*, *UPC2* and *HAP1* ranks did not vary with changes to ET. (Figure 6B, hexagons and crosses and Table 1).

**Table 1:** Comparison of single gene ranks for FL targets across increasing ETs

	***ERG11***		***ERG6***		***UPC2***		***HAP1***	
**ET Expt**	**Rank**	**Percentile**	**Rank**	**Percentile**	**Rank**	**Percentile**	**Rank**	**Percentile**
1 ET	2080	69	4686	30	588	91	859	87
2 ET	1752	74	4078	39	20	99.7	426	94
3 ET	1716	74	1461	78	483	93	291	96
4 ET	1835	73	852	87	39	99	56	99

Ranks for each FL gene target were reported for each ET. Ranks are the average of two replicate experiments. Rank percentile was computed as the percentage of genes with a rank as high or higher than the target gene. Rank percentiles ≥95 indicated significant perturbation of a gene as identified by SSEM-Lasso.

### Testing phase: Concentration changes have minimal to modest effects on FL gene target predictions

The second testing phase variable optimized was FL concentration. *S. cerevisiae* cells require ergosterol for normal aerobic growth [39], so it was necessary to determine a treatment concentration at which wild-type cells continued dividing with impaired ergosterol synthesis. FL dose-response curves and growth inhibitory values (GIs) were generated from in-house cell growth inhibition assays. FL dosing strategy was based on concentrations below the GI_50_ value – GI_0.5_, GI_5_, GI_10_, GI_20_, GI_30_ and GI_40_ – so that FL would not inhibit growth of more than 50% the cell population. Thus, gene perturbations were not considered direct results of cell death signalling pathways, but steady-state expression changes specific to FL. SSEM-Lasso results were evaluated within the same gene set and single gene analysis frameworks as the ET experiments.

**Figure 7:**
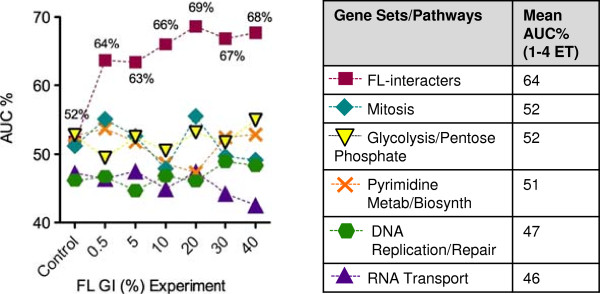
**Concentration effects on gene set (AUC%) analysis.** Areas under each sensitivity vs. rank threshold curve (ROC curve) for FL-interacters and orthogonal gene sets were converted to percentages (AUC%s) and plotted for each FL microarray concentration experiment. Cells were treated with FL concentrations that corresponded to increasing growth inhibitory percentages, GI%s (x-axis). Mean AUC%s (GI_0.5_ to GI_40_) for each gene set were computed and compared in the table. Larger AUC% values indicated better prediction of FL action on a gene set. AUC% values were the averages of two replicates.

Similar to the FL gene set trend for ET, higher FL treatment concentrations improved AUC% values. FL effects were obvious upon treatment, jumping from 51% for control to 64% for GI_0.5_ (Figure 7). AUC%s continued to improve as FL concentrations increased, up to GI_20_. These results demonstrated that SSEM-Lasso predicted FL action more accurately at treatment concentrations between GI_20_ and GI_40_, with AUC%s reaching 67-69% (Figure 7, squares). Orthogonal gene sets failed to show a similar trend as the FL-interacter gene set nor did they experience enhanced perturbation upon FL treatment. Average AUC% values remained below the FL-interacter gene set average of 64% across all FL concentrations (Figure 7). This was a positive indication that FL was specifically targeting genes associated with its activity.

**Figure 8:**
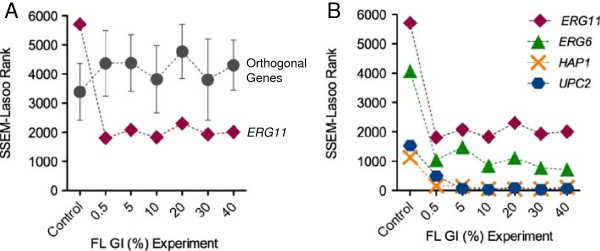
**Concentration effects on single gene (rank) analysis. (A)** SSEM-Lasso ranks of FL’s primary gene target, *ERG11* (diamonds), were compared to gene rank averages for six orthogonal genes, *MPS1*, *ADE13*, *TOP2*, *CDC9*, *PAB1* and *UBA1* (circles), across increasing FL concentrations. Error bars represent standard deviation for orthogonal genes. **(B)** SSEM-Lasso ranks of all FL targets, *ERG11* (diamonds), *ERG6* (triangles), *UPC2* (hexagons) and *HAP1* (crosses) versus concentration experiments. Cells were treated with FL concentrations that corresponded to increasing growth inhibitory percentages, GI%s (x-axis). Lower ranks indicated better prediction of FL action on an individual gene. All ranks are the averages of two replicates.

In general, FL single gene ranks were insensitive to changes in FL concentrations, just as they did not fluctuate significantly with changing ETs. At the onset of FL treatment, *ERG11* rank dropped from 5714 to 1812, a clear response to FL treatment. However over increasing treatment concentrations, *ERG11* ranks hovered modestly between 1800-2300 (ranks in the ~70^th^ percentile) with no noteworthy fluctuations (Figure 8A, diamonds and Table 2). Average ranks of the six orthogonal genes were predicted with SSEM-Lasso and proved unperturbed by FL. In addition, the orthogonal genes failed to experience the pronounced rank increase *ERG11* had at the onset of FL treatment (Figure 8A, circles), suggesting SSEM-Lasso was accurately predicting FL effects on its target.

**Table 2:** Comparison of single gene ranks for FL targets across increasing concencrations (GI%s)

	***ERG11***		***ERG6***		***UPC2***		***HAP1***	
**GI Expt**	**Rank**	**Percentile**	**Rank**	**Percentile**	**Rank**	**Percentile**	**Rank**	**Percentile**
Control	5714	14	4068	39	1543	77	1133	83
GI_0.5_	1812	73	1047	84	498	93	160	98
GI_5_	2081	69	1489	78	73	99	149	98
GI_10_	1835	73	852	87	39	99	56	99
GI_20_	2302	66	1119	83	93	99	105	98
GI_30_	1934	71	775	88	40	99	66	99
GI_40_	2007	70	721	89	74	99	116	98

Ranks for each FL gene target were reported for each concentration. Ranks are the average of two replicate experiments. Rank percentile was computed as the percentage of genes with a rank as high or higher than the target gene. Rank percentiles ≥95 indicated significant perturbation of a gene as identified by SSEM-Lasso.

*ERG6* followed a very similar concentration trend but with overall lower ranks than *ERG11* (Figure 8B, triangles). Transcription factors, *UPC2* and *HAP1*, had overall ranks below 200, or in 98^th^ to 99^th^ percentile (with the exception of *UPC2* for GI_0.5_ experiment). However, a pronounced trend over increasing concentrations was not observed (Figure 8B, hexagons and crosses and Table 2). These results were consistent with the ET results, wherein the FL-targeted transcription factors performed significantly better than the metabolic targets, *ERG11* and *ERG6*, and also were not as strongly affected by ET and concentration changes as *ERG11* and *ERG6*.

### Training phase: Input of microarray expression data and modification of the gene interaction network influences FL single gene target predictions

After exploring how experimental testing conditions influenced predictions of drug target predictions, focus shifted to the computational details of SSEM-Lasso’s training phase. Specifically, we generated modified training compendiums, or unique training phase variables, and examined how they altered the network’s gene-gene interaction “patterns” to improve final gene ranks. Previous SSEM-Lasso studies demonstrated that removal of experiments in the same project group from the training compendium boosted SSEM-Lasso performance, perhaps due to the reduction of artifactual effects caused by lab-specific experimental variables [12]. Here, we were interested in how the *addition* of microarray experiments related to the applied perturbation, FL treatment, affected predictions of FL gene targets.

Taking advantage of the *Saccharomyces* genome deletion collection [57,58], microarray expression data was obtained for three deletion strains related to FL mechanism of action, *erg11Δ/ERG11*, *erg6Δ, hem1Δ*[59], and one not linked to FL action, *spt3Δ*[60]. Expression data from the FL drug treatment that yielded the best predictions of *ERG11* perturbation (GI_10_ 4ET) was also included in training phase variations. First, we confirmed SSEM-Lasso identified the direct gene target for each deletion strain. In agreement with previous studies, SSEM-Lasso predicted all genetic deletion targets accurately (Table 3). We proceeded to individually add each experiment’s expression data into the original training compendium to generate five new training compendiums. Modified gene interaction networks were then inferred for each modified training compendium (Figure 4). Changes in gene rank (RC) between the original and modified training compendiums for FL targets, *ERG11*, *ERG6*, *ERG5*, and non-target *SPT3*, were subsequently determined.

**Table 3:** Gene ranks predicted by SSEM-Lasso for corresponding yeast genetic deletion microarray experiments

**Microarray experiment**	**Gene target**	**Rank with original compendium**	**Rank with modified compendium**
*erg11Δ/ERG11*^1^	*ERG11*	277	301
*erg6Δ*^1^	*ERG6*	1	1
*spt3Δ*^2^	*SPT3*	5	47
*hem1Δ*^3^	*ERG5*	40	41

^1^Christadore, L. Boston University. 2012.

^2^James, N., et al. *Genetics* 177:123 2007 [60].

^3^Protchenko O., et al. *Eukaryot. Cell* 7:859 2008 [59].

SSEM-Lasso gene ranks were determined for gene targets of genetic deletions and compound treatments. All microarray experiments were performed using Affymetrix Yeast Genome 98 gene chips, and data was RMA-normalized before processing with SSEM-Lasso algorithm. The original and modified training networks (modified with corresponding gene target expression data only) were used to determine ranks of target genes for each experiment. Raw data was obtained from published experiments (GEO) or conducted in in-house, as indicated in footnotes. Average ranks for duplicate experiments are shown.

**Figure 9:**
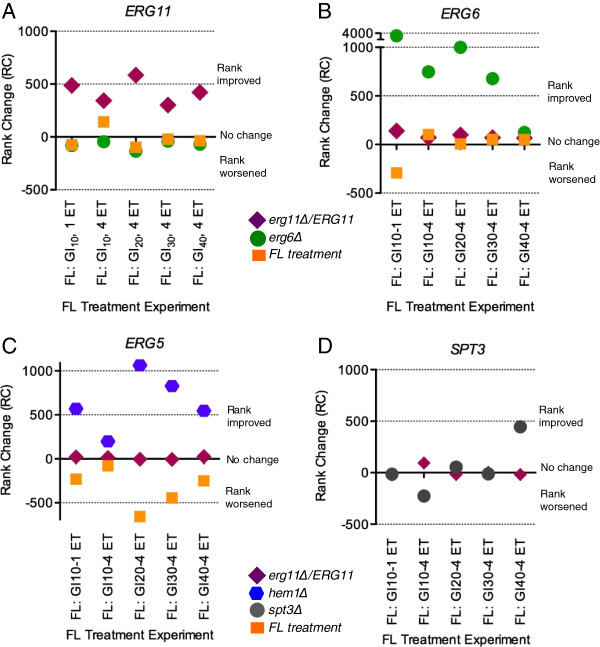
**Training phase variation effects on single gene (rank) predictions.** The modified training compendiums were used to predict ranks of FL-target genes, *ERG11*, *ERG6*, *ERG5*, and non-target gene, *SPT3*, in five representative FL treatment experiments. First, gene ranks for 2 replicate experiments were averaged. Next, ranks from the original training compendium were subtracted from ranks derived from the modified training compendium, yielding rank changes, or RCs. Finally, RCs (y-axis) were plotted for five representative FL treatment experiments (x-axis) for each gene: **(A) ***ERG11*, **(B) ***ERG6*, **(C) ***ERG5*, and **(D) ***SPT3*. Positive RCs signified the gene rank improved with the addition of the corresponding deletion experiment data to the compendium. An RC of 0 indicated no change. A negative RC indicated rank increased or worsened.

Graphs in Figure 9A-D display five representative FL treatment experiments on the x-axis and RC values for *ERG11*, *ERG6*, *ERG5* or *SPT3* on the y-axis. These results showed that each modification to the training compendium induced changes to the gene network that were unique to the deletion strain’s corresponding target gene. *ERG11*, *ERG6,* and *ERG5* ranks dropped, or improved, *only* with the addition of expression data from *erg11Δ/ERG11*, *erg6Δ,* and *hem1Δ*, respectively. In contrast to the addition of genetic deletions, inclusion of expression data from the FL treatment experiment did not impact network interactions to alter *ERG11*, *ERG6* and *ERG5* ranks. In most cases, ranks stayed the same or worsened with the addition of FL drug treatment expression data (Figure 9A-C, Tables 4, 5 and 6).

**Table 4:** **Comparison of rank changes (RCs) for *ERG11 *in network training phase**

	***ERG11***			
	***+ Δerg11/ERG11***		***+ Δerg6***	**+ FL**
**Expt analyzed**	**Rank change (RC)**	**Percentile**	**Rank change (RC)**	**Rank change (RC)**
FL_GI10_, 1DT	488	97	−82	−75
FL_GI10_, 4DT	344	92	−45	142
FL _GI20_, 4DT	585	96	−136	−98
FL _GI30_, 4DT	301	88	−41	−21
FL _GI40_, 4DT	421	94	−70	−37

RCs were reported for *ERG11* across 5 FL treatment experiments, under 3 different training compendiums: + *Δerg11/ERG11*, + *Δerg6*, and + FL treatment. Percentile of RC was computed as the percentage of genes with a RC as high or higher than the target gene. RC percentiles ≥95 indicated significant perturbation as identified by SSEM-Lasso.

**Table 5:** **Comparison of rank changes (RCs) for *ERG6 *in network training phase**

	***ERG6***				
	***+ Δerg6***		***+ Δerg11/ERG11***		**+ FL**
**Expt analyzed**	**Rank change (RC)**	**Percentile**	**Rank change (RC)**	**Percentile**	**Rank change (RC)**
FL_GI10_, 1DT	3795	100	139	73	−292
FL_GI10_, 4DT	748	98	71	43	102
FL _GI20_, 4DT	999	99	99	57	6
FL _GI30_, 4DT	677	97	70	47	54
FL _GI40_, 4DT	123	48	67	45	52

RCs were reported for *ERG6* across 5 FL treatment experiments, under 3 different training compendiums: + *Δerg6*, + *Δerg11/ERG11*, and + FL treatment. Percentile of RC was computed as the percentage of genes with a RC as high or higher than the target gene. RC percentiles ≥95 indicated significant perturbation as identified by SSEM-Lasso.

**Table 6:** **Comparison of rank changes (RCs) for *ERG5 *in network training phase**

	***ERG5***			
	***+ Δhem1***		***+ Δerg11/ERG11***	**+ FL**
**Expt analyzed**	**Rank change (RC)**	**Percentile**	**Rank change (RC)**	**Rank change (RC)**
FL_GI10_, 1DT	569	97	23	−230
FL_GI10_, 4DT	199	75	16	−80
FL_GI20_, 4DT	1064	97	−5	−656
FL_GI30_, 4DT	828	95	−6	−444
FL_GI40_, 4DT	547	90	26	−251

RCs were reported for *ERG5* across 5 FL treatment experiments, under 3 different training compendiums: + *Δhem1*, + *Δerg11/ERG11*, and + FL treatment. Percentile of RC was computed as the percentage of genes with a RC as high or higher than the target gene. RC percentiles ≥95 indicated significant perturbation as identified by SSEM-Lasso.

*ERG11* RCs for all FL treatment experiments dropped substantially when *ERG11* deletion expression data was added to the training compendium. *ERG11* ranks were in the 97^th^, 92^th^, 96^th^, 88^th^, or 94^th^ percentile of RCs for each of the five experiments (Table 4). Inclusion of *ERG6* deletion expression data into the training compendium also improved prediction of *ERG6* perturbations across all FL experiments. In fact, inclusion of the haploid *erg6Δ* experiment to the training compendium produced a more pronounced drop in *ERG6* ranks than the heterozygous *erg11Δ/ERG11* experiment did for *ERG11* ranks. *ERG6* RC percentiles were in the range of 97 to 100, with the exception of the FL_GI40_, 4DT experiment (Table 5). Addition of *hem1Δ* expression data also improved *ERG5* ranks for all FL treatment experiments, and *ERG5* RCs were in the 97^th^, 75^th^, 97^th^, 95^th^, or 90^th^ percentiles (Table 6). *ERG5* rank improvements were similar in magnitude and direction to those of *ERG11*. This result suggested that *ERG11* and *ERG5* were similarly affected by FL, and also by heme depletion, supporting their synergy along the ergosterol biosynthesis pathway and FL’s interactions with their CYP heme centers.

To test if network changes induced by *erg11Δ/ERG11*, *erg6Δ* and *hem1Δ* were specific to FL, we analyzed whether haploid *spt3Δ* expression data improved *SPT3* ranks for the same FL experiments. Spt3p, a subunit of SAGA-type histone acetyltransferase complex, is not specifically targeted by FL. Indeed, inclusion of *spt3Δ* expression data did not significantly alter *SPT3* rank across the representative FL experiments; three of the ranks increased while two widely fluctuated (Figure 9D, Table 7).

**Table 7:** **Comparison of rank changes (RCs) for *SPT3 *in network training phase**

	***SPT3***		
	***+ Δspt3***		***+ Δerg11/ERG11***
**Expt analyzed**	**Rank change (RC)**	**Percentile**	**Rank change (RC)**
FL_GI10_, 1DT	−15	n/a	−23
FL_GI10_, 4DT	−226	n/a	95
FL_GI20_, 4DT	55	63	−17
FL_GI30_, 4DT	−12	n/a	4
FL_GI40_, 4DT	445	98	−18

RCs were reported for *SPT3* across 5 FL treatment experiments, under 3 different training compendiums: + *Δspt3* and + *Δerg11/ERG11*. Percentile of RC was computed as the percentage of genes with a RC as high or higher than the target gene. RC percentiles ≥95 indicated significant perturbation as identified by SSEM-Lasso.

### Training phase: A further application of network variations to nocodazole gene target predictions

We explored if these findings were applicable to nocodazole (NOC), an antimitotic benzimidazole drug that destabilizes microtubules and causes cell cycle arrest in mitosis [48]. *TUB1* is one of two functional genes (the other is *TUB3*) that encodes for α-tubulin, an essential protein of the α,β-tubulin heterodimer [49]. α,β-tubulin polymerizes into microtubules, which are critical components of the mitotic and meiotic spindles and essential for cell division [49]. We hypothesized that addition of microarray expression data from a *tub1Δ/TUB1* deletion strain could potentially improve *TUB1* rank in NOC treatment experiments.

All experiments were performed in BY4741 strains using the same media and conditions that were used in FL experiments. We determined NOC treatment concentrations using dose-response curves and growth inhibitory values (GIs) as we did for FL. Studies have shown that *S. cerevisiae* haploid cells treated with NOC (6 to 15 μg/mL, or 20 to 50 μM) rapidly disassemble microtubules in the majority of the cell population, resulting in mitotic arrest with intact nuclei [48,50]. We chose a concentration slightly lower, 16 μM, which corresponded to GI_10_, and incubation intervals of 3 and 4 ETs.

Interestingly, SSEM-Lasso did not predict *TUB1* perturbation as well as it did for *ERG11* with the original training set. Still, upon addition of *TUB1* deletion expression data, ranks dropped by over 300, and *TUB1* RCs were in the top 98% and 97% of all RCs for GI_10_, 3ET and GI_10_, 4ET experiments, respectively (Table 8).

**Table 8:** Nocodazole study results in network training phase

	***TUB1***		
	***+ Δtub1/TUB1***		
**Expt analyzed**	**Rank**	**RC**	**Percentile**
NocGI_10_, 3DT	4283	333	98
NocGI_10_, 4DT	4640	371	97

Two nocodazole (NOC) treatment experiments were performed and RCs of a NOC target gene, *TUB1*, were evaluated. Ranks listed in table were the single values each obtained from one treatment experiment.

## Discussion

### Impact of testing phase variations on SSEM-Lasso predictions of gene sets

SSEM-Lasso was originally developed to identify a directly targeted gene of a genetic or drug perturbation [12]. We sought to expand the algorithm’s utility by testing its ability to predict drug perturbations over several biological conditions *and* on a set of biologically-related genes, rather than on a single gene target. This was the first study that analyzed SSEM-Lasso output using ROC curves and corresponding AUC values for a group of predefined genes.

FL treatment of *S. cerevisiae* results in inhibition of lanosterol demethylation and subsequent depletion of ergosterol and accumulation of toxic methylated sterol precursors. This results in cell membrane damage and impaired fatty acid and lipid biosyntheses [35,37]. FL coordinates to Erg11p’s heme iron impairing cytochrome-related processes, such as mitochondrial respiration (reviewed in Parks, et al. 1995 [36]). We created a gene set based on these biological processes, called FL-interacters. Experimental variations in ET and concentration manifested perturbations that followed a clear trend and were specific to the FL-interacters gene set. An ET of at least four cell doublings in the presence of drug proved optimal for accurate prediction of FL-interacters (Figure 5) Concentration changes did not exert as strong of an effect as ET, and any concentration between GI_10_ and GI_40_ proved optimal to induce target-gene perturbations (Figure 7). Our results demonstrated that experimental “input” variables could be successfully optimized to identify a set of related genes targeted by a drug. Our methodology could therefore provide an experimental platform for future studies aimed at predicting drug targets at the multi-gene or pathway level.

In principle, given the training/testing paradigm common to the core of statistical and machine learning methods in general, the spirit of the analyses carried out in this study may be similarly used in exploring the sensitivity of other methods of drug-target prediction methods. However, researchers should be aware that optimal experimental conditions determined in this study are exquisitely linked to SSEM-Lasso and FL. Drugs with different modes-of-action, potencies, and potential for off-target effects may have dissimilar effects on gene expression under these reported experimental conditions. Further, FL effects could manifest dissimilar genetic perturbations when analyzed with a new statistical model. Finally, depending on the algorithm or supervised learning method, results may be more or less sensitive to experimental changes than those reported with SSEM-Lasso. Therefore, the approach would need to be adapted to the specifics of the measurements at hand, which have tended to vary in the literature (e.g., here we utilize only microarray expression profiles). Nevertheless, this study provides a good starting point for researchers to obtain accurate drug target predictions using microarray technology.

### Impact of testing phase variations on SSEM-Lasso drug target predictions at the single gene level

In our study, SSEM-Lasso accurately predicted FL single gene targets upon drug treatment, yet experimental variables did not dramatically affect biological outcomes. With the exception of *ERG6* (which showed a distinct rank improvement after two cell cycles of FL treatment), *ERG11*, *UPC2*, and *HAP1* ranks did not significantly fluctuate over varying treatment conditions.

 FL is known to target *ERG11* (primary), *ERG6*, *UPC2*, and *HAP1*, so these genes were tracked to evaluate optimal experimental conditions. Unlike gene set predictions, SSEM-Lasso prediction of *ERG11* perturbation was relatively unchanging over multiple testing phase modifications (Tables 1 and 2). These results demonstrated that changes in *ERG11* expression caused by changes in ET and concentration were not significantly detected by SSEM-Lasso above the background gene-gene interaction “patterns” in the network. *ERG6* expression, on the other hand, responded to longer FL exposure times, with four ETs yielding an optimal *ERG6* rank in the top 87% of ranked genes (Table 1). Furthermore, *ERG6* ranked consistently lower than *ERG11* across all FL concentration experiments (Figure 8B, Table 2). *ERG6* encodes for Δ [24]-sterol C-methyltransferase, an enzyme downstream of Erg11p that synthesizes fecosterol, an important precursor to ergosterol (Figure 2). FL effects may have manifested more prominently on *ERG6* than on *ERG11* because of the cell’s reliance on Erg6p for pleiotropic cellular processes, including membrane rigidity and permeability, genetic transformation, conjugation and tryptophan uptake [42,43]. Furthermore, Erg6p places a high demand on the cell for metabolic energy, 12-14 ATP equivalents, to perform transmethylation reactions [36]. Another reason *ERG6* may have ranked lower than *ERG11* involves the unnatural accumulation of lanosterol and/or the depletion of ergosterol as a result of Erg11p inhibition by FL. Disruption of later stage ergosterol biosynthesis enzymes, like Erg6p, has been attributed to the build up of reactant metabolites along the ergosterol pathway [61]. Furthermore, genome-wide microarray studies have found the most responsive genes to ketoconaozle function downstream of *ERG11*, suggesting their induction is induced by ergosterol depletion [61].

### Metabolic vs. transcription factor gene target predictions

A different trend, in comparison to metabolic enzymes *ERG11* and *ERG6*, was observed for transcription factors, *UPC2* and *HAP1*. Upc2p induces the transcription of ergosterol biosynthesis genes upon sterol depletion [44,45]. Previous microarray studies in *S. cerevisiae* had identified induced *UPC2* expression in response to ketoconaozle [62]. This result was consistent with the FL-induced *UPC2* perturbation observed in this study. SSEM-Lasso pinpointed *UPC2* dysregulation by FL, with ranks in the 99^th^ percentile at four ETs and a range of concentrations (Tables 1 and 2). Depending on the presence of heme, Hap1p can bind the promoters of genes to activate or repress their expressions for aerobic or hypoxic growth, such as the CYP450 enzymes, *ERG11* and *ERG5*[48]*.* Hence, *ERG* gene expressions are highly dependent on *HAP1* gene interactions and the aerobic state of the cell. Additionally, Hap1p represses transcription of its own gene by at least 20-fold in a heat shock protein-dependent manner [63]. Ranks for *HAP1* were comparable to *UPC2* ranks, all performing significantly better than other genes at three and four ETs at higher FL concentrations (Tables 1 and 2). To factor perturbations better than metabolic enzymes in the context of a drug treatment.

We discovered that the type of molecular target – metabolic enzyme or transcription factor, upstream or downstream – can play a large role in the overall performance of the algorithm.

### Training phase variables significantly improve single gene target predictions

The goal of SSEM-Lasso’s training phase was to infer a gene interaction network that filtered out gene-gene regulatory “patterns” so that genes experiencing a true additive shift in their transcript signals emerged above the gene network background. In this study, modified training compendiums, the training phase “variables”, were created by adding of new gene deletion data to the training compendium. Each modified training compendium shifted the network’s internal gene regulatory influences so that SSEM-Lasso more accurately predicted single gene targets. This was evidenced by significant rank decreases for *ERG11*, *ERG6* and *ERG5* upon addition of *erg11Δ/ERG11, erg6Δ*, and *hem1Δ* data, respectively, to the training compendium (Figure 9A-C, Tables 4, 5 and 6). The *erg6Δ* haploid deletion induced the most pronounced decrease in *ERG6* rank predictions. This could be a result of more potent and specific transcriptional effects of the *erg6Δ* knockout compared to the partial knockout, *erg11Δ/ERG11*, or the *hem1Δ* strain, which was shown to induce widespread transcriptional effects on many cellular processes [59].

Additional studies involving NOC treatment and *TUB1* expression supported these FL findings. Rank changes for NOC target, *TUB1*, dropped significantly when the original training compendium was modified with *tub1Δ/TUB1* deletion data (Table 8). Only one replicate experiment of two NOC conditions, GI_10_ 3 DT and GI_10_ 4 DT, was performed for this drug target study, which is a limitation to the analysis. Finally, rank improvements of a non-FL-targeted gene, such as *SPT3*, were not manifested with addition of corresponding haploid *spt3Δ* expression data to the compendium (Figure 9D). When taken together, these results indicated that gene targets and the modified training compendium should be specific to the drug under investigation in order to improve SSEM-Lasso’s performance. Still, further training phase variations concerning other drug treatments and deletion strains, and including more replicates, are necessary to assess the applicability of this strategy to various drug-target predictions.

It was initially hypothesized that addition of FL treatment expression data to the training compendium could also minimize background gene-gene interactions specific to FL’s effects. This was not the case, as inclusion of the FL experiment, GI_40_, 4ET, modified gene regulatory “patterns” in a negative manner, increasing ranks for FL targets. This led to the conclusion that genetic knockouts produced more distinct and local effects on the network as opposed to “noiser” and potentially off-target perturbations induced with an exogenous compound. Additionally, *S. cerevisiae* is susceptible to “neighboring gene effects”, whereby the phenotype of a particular strain (e.g., deletion strain) could be due to the effect the perturbation exerts not on the target gene, but on an “adjacent” gene [64]. With around 10% of all yeast genes experiencing this type of influence [64], it was possible that there were too many “neighboring” expression changes induced by a foreign compound, and thus “noise” was enhanced and direct FL gene targets were pushed further into the background.

The training phase variables caused dramatic improvements in SSEM-Lasso performance compared to the minor rank fluctuations observed for experimental testing variations. Thus, when examining a drug’s potential effects using microarray data and our methodology, the experimental conditions played a more important role in predicting a gene set. On the other hand, fine-tuning of the computational variable, i.e. the gene interaction network, proved more critical for accurate predictions of single gene drug targets.

### SSEM-Lasso utility for the prediction of unknown drug targets

These results suggest a unique, two-stage approach to predict an unknown drug target using genome-wide microarray data and a network-inference model, such as SSEM-Lasso. First, detection of one or more perturbed gene sets should hone in on one or more biological pathways affected by the drug in question. In the case of SSEM-Lasso, these pathways were set *a priori* by the experimentalist, permitting a more focused analysis of drug-induced effects. Once a target pathway is elucidated, genetic deletion data specific to genes along the target pathway can be added to the training data. Single gene results can then be analyzed under different network training conditions and compared to arrive at more accurate drug gene target predictions. Genes experiencing greater rank changes with the addition of their respective genetic deletion data to the training compendium can be considered more likely candidate drug targets.

## Conclusions

The goal of this study was to improve our understanding of the interaction between biological testing and computational training variables in order to produce more accurate predictions of drug action at the transcriptional level. Previously, the network-inference model, SSEM-Lasso, predicted molecular targets of genetic mutations more accurately than drug treatments. We discovered a two-stage approach that addressed this issue and outlined improved experimental and computational conditions for predictions of first, drug pathway and second, single gene targets. Experimentally, the duration cells are in contact with a compound and the changes in compound concentration do not have a dramatic effect on single gene targets. However, these variables must be optimized for more accurate target predictions at the gene set/pathway level. Computationally, addition of specific biologically-motivated expression data to the interaction network can influence the gene regulatory effects in a manner that better resolves perturbations at the single gene level. With these variables in mind, SSEM-Lasso, and by extension, similar computational methods, can be a tremendously useful tool for therapeutic discovery when implemented under the appropriately informed testing and training conditions.

## Methods

### Yeast strains and treatment conditions

All FL treatment experiments maintained constant background, growth conditions, and mRNA preparation and hybridization procedures. The wild-type *S. cerevisiae* strain derived from BY4741 (*MAT*a *his3Δ1 leu2Δ0 met15Δ0 ura3Δ0*) was used for all FL treatments. This cell line has been used for systematic sequencing and deletion projects in which open reading frames were replaced by kanamycin cassettes (KanMX) to generate haploid and heterozygous knockouts [44]. Heterozygous knockout strains, *ergllΔ/ERG11* and *tublΔ/TUB1* in a BY4743 background (*MATa/MATα his3Δ1/his3Δ1 leu2Δ0/leu2Δ0 lys2Δ0/+ met15Δ0/+ ura3Δ0/ura3Δ0*), and haploid strain, *erg6Δ* in a BY4741 background were generated by the *Saccharomyces* genome deletion project [45] (Invitrogen) and used for genetic deletion microarray experiments. Haploid strain, *hem1Δ* in a BY4742 background (*MAT***α***his3Δ1 leu2Δ0 lys2Δ0 ura3Δ0*) was cultured under heme depletion conditions and microarray hybridization was performed according to Protchenko et al., 2008 [46].

Yeast growth is typically measured in cell population doubling times, determined by turbidity [65]. Consequently, a treatment collection time point, or exposure time (ET), was defined as the time it took a cell population to double in the presence of FL. ETs were longer than a typical 90-minute doubling time for wild-type *S. cerevisiae* cells at 30°C, an anticipated result attributed to FL fungistatic activity.

### Determination of treatment concentrations

To determine FL and NOC growth inhibitory value (GI), a single colony of wild-type cells was inoculated into 10 mL YPD media (1% yeast extract, 2% bacto-peptone, 2% dextrose) overnight, diluted with YPD medium to give an OD_600_ of 0.005, and pipetted (200 μl) into a flat-bottom 96-well plate. Serial dilutions of FL (Sigma) or NOC (Calbiochem) were prepared in 100% DMSO and added (5 μL) to cells to obtain final concentrations of 200 μM - 10 μM (for FL) and 60 - 10 μM (for NOC). Each concentration was represented 6x on a single plate. The plates were incubated at 30°C overnight and the OD_600_ of control and treated wells were determined. Control absorbance values were normalized to 0% inhibition. Dose-response curves were generated by plotting the % growth inhibition (final) versus the log_10_ (drug concentration). GI percentages (0.5, 5, 10, 20, 30, 40) were then determined with GraphPad using the four parameter fit model.

### Genome-wide microarrays

A single colony of wild-type or deletion yeast strains was inoculated into 10 ml YPD media overnight, then diluted with YPD medium to give an OD_600_ between 0.08 and 0.1. G418 was used for KanMX selection conditions of the deletion strains (final concentration 200 mg/L). Cells were immediately treated with the appropriate concentration of FL, NOC, or DMSO (final concentration 1 v/v % DMSO), incubated at 30°C with shaking (250 rpm), and collected at mid-log growth phase for one, two, three, or four ETs. Cells were harvested by centrifugation at 500 x g, 5 minutes, room temperature, flash frozen and stored at -80°C. Total RNA was isolated using the acid phenol chloroform method. Briefly, cell pellets were thawed, re-suspended in lysis buffer, and RNA was extracted with hot acid phenol:chloroform (Fisher). After three extractions, the supernatant, containing RNA, was added to 100% cold ethanol, and RNA was allowed to precipitate at -20°C for 4-6 hours. RNA was pelleted and washed with 70% ethanol before dissolving in DEPC-treated water. Poly(A)^+^ RNA was next isolated using Oligotex mRNA kit (Qiagen Inc.) and amplified and hybridized to Affymetrix YG S98, except only 25 ng of mRNA was used. Raw expression data was RMA-normalized and processed with SSEM-Lasso.

### Establishing gene sets

Relationships between genes in a gene set were based on published literature and biological pathway and yeast genome databases, Kyoto Encyclopedia of Genes and Genomes (KEGG) and Gene Ontology (GO). Each gene set contained between 72 and 74 genes. See Additional files for specific genes.

### SSEM-Lasso algorithm and predictions of gene targets

Methods of Cosgrove et al., 2008 [12] were adopted to identify gene perturbations using Lasso regression in a sparse simultaneous equation model (SSEM-Lasso). We briefly sketch the key elements of this approach here; refer the reader to Cosgrove et al., 2008 [12] for full details. With SSEM-Lasso, the mean level of gene expression from a single gene is described as a function of two elements (1) the gene expression of all other genes in a network and (2) an external perturbation parameter. The notion of a “targeted gene” refers to an external perturbation to the mean mRNA level of a gene that cannot be explained by gene-gene interactions alone. For p genes and n observations, the model can be written as: *Y* = *BY* + Φ + *E*, where *Y* is a p by n matrix of gene transcript measurements (for p genes in n samples), *B* is a p by p matrix of gene-gene interaction effects whose diagonal elements are fixed to zero (gene interaction network/matrix), Φ is a p by n matrix of external perturbations (derived from the experiment being tested) and *E* is a p by n matrix of random noise assumed Gaussian with zero mean.

Note that the model used in SSEM-Lasso is an auto-regressive model, with the variable *Y* serving as both response and predictor. Auto-regressive models have a long history in traditional time series and spatial data analysis [66], and in recent years have proven popular for network-based modelling as well [67] (See Kolaczyak, 2009 Ch7.3). The manner in which we write the model above is a standard and concise representation [66]. In this form, the relationship among the gene expression is summarized (1) across all microarrays (i.e., it is a multivariate statistical representation, involving the entire matrix *Y*, rather than a single column), and (2) at the level of the joint marginal distribution within each microarray (i.e., it involves the full columns in *Y*, rather than just their individual elements). However, at the level of an individual measurement in *Y*, say single gene k in sample i, denoted as *y*_*ki*_, the model may be shown to specify that the conditional distribution of *y*_*ki*_, given all *other* genes j ≠ k in experiment i, denoted as *y*_*ji*_, is of the form

$$y_{\mathrm{ki}}=\sum_{j\neq K}B_{\mathrm{kj}}y_{\mathrm{ji}}+\varphi_{\mathrm{ki}}+e_{\mathrm{ki}}$$

That is, conditionally, the expression of any one gene is modelled as a linear combination of that of the others, plus a possible perturbation, plus a noise term [12]. Thus, this type of model is a natural way of capturing the notion of the expression levels of each gene being influenced by the expression levels all other genes. Additionally, this conditional form shows why a regression-based strategy is natural for estimating the unknown parameters in *B*.

As in Cosgrove et al., 2008, the method was implemented in two steps. First, the original training compendium of RMA-normalized Affymetrix data was used to infer the gene-gene interaction network B. This training compendium consisted of 1039 Affymetrix YG S98 GeneChips, representing 465 experimental conditions [12]. A simpler, simultaneous equation model was assumed by setting Φ to zero and estimating B row-by-row using a sparse regression technique.

Lasso regression is a form of penalized regression, in which the standard least squares goodness-of-fit criterion is augmented with an additional term capturing the sum of the absolute values of all regression coefficients in the model. Such penalties, developed and studied extensively over the past two decades, are known to encourage sparse models and are particularly useful in contexts (such as the current one) in which a relatively small number of variables (i.e., genes) need to be selected from among a very large number. See Cosgrove et al., 2008 [12] for additional details on implementation of the Lasso methodology, and [68], for a formal characterization of the performance of SSEM-Lasso (including the expected accuracy of the Lasso-based regression), both theoretical and under simulation.

In the second step of the SSEM-Lasso method, using the estimate of B resulting from the first step, an outlier analysis of the residuals is conducted: ${\hat{r}}^{\mathrm{pert}}=y^{\mathrm{pert}}-By^{\mathrm{pert}}$, where y^pert^ is a p x 1 vector of expression values across p genes in a single experiment (in our case a FL experiment). The residual, ${\hat{r}}^{\mathrm{pert}}$ is a combination of the external influence *Φ*^pert^ of the perturbation and noise. Residuals were then ranked by their absolute values for all annotated yeast genes (1-6681). Genes with low ranks (and thus high residuals) were genes that SSEM-Lasso distinguished from the gene network background and were considered potential targets of the applied perturbation.

### Modification of the training compendium

For FL training phase modifications, raw expression data from two, individual microarray experiments performed under identical conditions (e.g. two replicates of an Affymetrix GeneChip from an *erg11Δ/ERG11* heterozygous mutant experiment) were RMA-normalized. Expression data were input into the training compendium as a single experiment file. A new, distinct training network was inferred as described above. In this way, the gene interaction matrix acted as the variable for the training phase modification experiments. RMA-normalization for all modified training compendiums was done in one step and included GeneChips from the original training compendium [12] plus additional experiments: *erg11Δ/ERG11*, *erg6Δ*, *hem1Δ* (GSM241150 and GSM241150), *spt3Δ* (GSM239658 and GSM239659), Fluconazole treatment, *rpl7aΔ* (GSM217617 and GSM217618) and Pterostilbene treatment (GSM266726 and GSM266728).

### Determining gene ranks, rank changes, and percentiles

After implementing SSEM-Lasso, gene ranks for all FL treatment experiments were obtained and averaged for each experimental condition (see Background and Figure 1). Gene ranks for NOC experiments were single experiments from each condition. All ranks fell between 1 and 6681, which were the total number of genes in the compendium. The percentile of a target gene in the testing phase was computed by dividing the total number of genes with ranks less than the target gene by the total number of genes (6681). Rank changes (RCs) were computed for the training phase by subtracting the rank obtained with the modified training compendium from the rank obtained with the original compendium. The RC percentile of a target gene in the testing phase was similarly computed.

## Competing interests

The authors declare that they have no competing interests.

## Authors’ contributions

LC conducted all biological assays and computational and qualitative analyses of SSEM-Lasso results. LC wrote the manuscript. LP processed microarray expression data through SSEM-Lasso, generated ROC curves, and contributed to the statistical methods and explanations in the manuscript. EK and SES conceived this study as a progression of a previous SSEM-Lasso study, conducted by Elissa Cosgrove (EC). EC and Yingchun Zhou developed SSEM-Lasso for gene target identification studies. All authors read and approved the final manuscript.

## Supplementary Material

Additional file 1 **(Previous_Predictions_SSEMLasso.pdf) - Previous SSEM-Lasso predictions of genetic and drug gene target perturbations.** SSEM-Lasso gene ranks are listed for gene targets of genetic deletions and bioactive compound/drug treatments. All microarray experiments were performed using Affymetrix Yeast Genome 98 gene chips, and data was RMA-normalized before processing with SSEM-Lasso. Experiments cited were published, annotated or conducted in in-house. Average ranks for experiments that provided replicates are shown. Click here for file

Additional file 2 (FL-interacters_Gene_Set.pdf) - FL-interacters Gene Set. Click here for file

Additional file 3 (DNA_Gene_Set.pdf) - Orthogonal Gene Set: DNA replication and repair. Click here for file

Additional file 4 (Pyrimidine_Gene_Set.pdf) - Orthogonal Gene Set: Pyrimidine biosynthesis and metabolism. Click here for file

Additional file 5 (RNA_Gene_Set.pdf) - Orthogonal Gene Set: RNA transport. Click here for file

Additional file 6 (Glyc_Pent_Gene_Set.pdf) - Orthogonal Gene Set: Glycolysis and pentose phosphate. Click here for file

Additional file 7 (Mitosis.pdf) - Orthogonal Gene Set: Mitosis. Click here for file
