# Supplementary material for: Improvement of experimental testing and network training conditions with genome-wide microarrays for more accurate predictions of drug gene targets
Source: BMC Syst Biol. 2014 Jan 20;8:7. doi: 10.1186/1752-0509-8-7 (PMC3911882; doi:10.1186/1752-0509-8-7)
Supplement: Additional file 1 — (Previous_Predictions_SSEMLasso.pdf) - Previous SSEM-Lasso predictions of genetic and drug gene target perturbations. SSEM-Lasso gene ranks are listed for gene targets of genetic deletions and bioactive compound/drug treatments. All microarray experiments were performed using Affymetrix Yeast Genome 98 gene chips, and data was RMA-normalized before processing with SSEM-Lasso. Experiments cited were published, annotated or conducted in in-house. Average ranks for experiments that provided replicates are shown. [file 1752-0509-8-7-S1.pdf]

GENETIC PERTURBATIONS				CHEMICAL PERTURBATIONS			
**Gene Target**	**Deletion Strain**	**Rank**	**Reference**	**Gene Target**	**Compound Treatment**	**Ran k**	**Reference**
ASF1	*asf1Δ*	6	Zabaronick & Tyler, *Mol. Cell. Biol.* **25**, 652-660 (2005)	*ERG28*	Amphotericin B	1630	Agarwal et al., *J. Biol. Chem*. **278**, 34998-35015 (2003)
AZF1	*azf1Δ*	13	Slattery et al., *Euk. Cell* **5**, 313-320 (2006)	*TOP1*	Camptothecin	864	Dominguez Boston Univ.
CDC21	*cdc21Δ/ CDC21*	104	Christadore Boston Univ. (2009)	*FKS1*	Caspofungin	43	Agarwal et al., *J. Biol. Chem*. **278**, 34998-35015 (2003)
DCP1	*dcp1Δ*	1	Feng et al., *Mol. Cell* **12**, 296-299 (2003)	*GSC2*	Caspofungin	766	Agarwal et al., *J. Biol. Chem*. **278**, 34998-35015 (2003)
ERG6	*erg6Δ*	1	Christadore Boston Univ. (2009)	*CIN5*	Cisplatin	5515	Birrell et al., *PNAS* **99**, 8778-8783 (2002)
FKS1	*fks1Δ*	1	Bulik et al., *Euk. Cell* **2**, 886-900 (2003)	*CTR1*	Cisplatin	3650	Birrell et al., *PNAS* **99**, 8778-8783 (2002)
FUM1	*fum1Δ*	1	Kokko et al., *Intl. J. Cancer* **118**, 1340-1345 (2006)	*IXR1*	Cisplatin	2147	Birrell et al., *PNAS* **99**, 8778-8783 (2002)
HHT2	*hht2Δ*	1	Yu et al., *J. Biol. Chem.* **7;281**, 9755-9764	*RPL28*	Cycloheximide	1134	Dominguez Boston Univ.
HSL1	*hsl1Δ*	4	Dominguez Boston Univ. (2006)	*ERG11*	Fluconazole	2604	Dominguez Boston Univ.

NAM7	*nam7Δ*	1	Feng et al., *Mol. Cell* **12**, 296-299 (2003)	*CDC21*	5-Fluorocytosine	4801	Agarwal et al., *J. Biol. Chem*. **278**, 34998-35015 (2003)
RPD3	*rpd3Δ*	2	Sabet et al., *Mol. Cell Biol.* **24**, 823-33 (2004)	*FCY1*	5-Fluorocytosine	2220	Agarwal et al., *J. Biol. Chem*. **278**, 34998-35015 (2003)
RPL7a	*rpl7Δ*	1	Komili S et al., *Cell* **131**, 557-71 (2007)	*FUR1*	5-Fluorocytosine	199	Agarwal et al., *J. Biol. Chem*. **278**, 34998-35015 (2003)
SGS1	*sgs1Δ*	7	Fry et al., *Mech. Ageing Dev*. **124**, 839-46 (2003)	*ERG11*	Ketoconazole	2038	Agarwal et al., *J. Biol. Chem*. **278**, 34998-35015 (2003)
SPT3	*spt3Δ*	5	James et al., *Genetics* **177**, 123-35 (2007)	*CHS3*	GlcNAc	3966	Bulik et al., *Euk. Cell* **2**, 886-900 (2003)
SPT10	*spt10Δ*	29	Mendiratta et al., *J. Biol. Chem.* **281**, 7040-7048 (2006)	*TUB1*	Nocodazole	3536	Dominguez Boston Univ.
TOP2	*top2Δ/ TOP2*	23	Christadore Boston Univ.	*TUB2*	Nocodazole	2524	Dominguez Boston Univ.
TOR2	*tor2Δ*	46	Mulet et al., *J. Biol. Chem.* **281**, 33000-7 (2006)	*TOR1*	Rapamycin	6530	Dominguez Boston Univ.
YHB1	*yhb1Δ*	1	Bourges et al., Array Express (2006)	*TOR2*	Rapamycin	3709	Dominguez Boston Univ.
				RPB10	Thiolutin	875	Guan et al., *PLoS Genetics* **2**, e203.
