# Supplementary material for: Improvement of experimental testing and network training conditions with genome-wide microarrays for more accurate predictions of drug gene targets
Source: BMC Syst Biol. 2014 Jan 20;8:7. doi: 10.1186/1752-0509-8-7 (PMC3911882; doi:10.1186/1752-0509-8-7)
Supplement: Additional file 2 — (FL-interacters_Gene_Set.pdf) - FL-interacters Gene Set. [file 1752-0509-8-7-S2.pdf]

Gene Name	ORF Name	Gene Name	ORF Name
ACC1	YNR016C	*HAP1*	YLR256W
ARE1	YCR048W	*HEM1*	YDR232W
ARE2	YNR019W	*HES1*	YOR237W
ATG26	YLR189C	*HFA1*	YMR207C
AUS1	YOR011W	*HMG1*	YML075C
BTS1	YPL069C	*HMG2*	YLR450W
CCP1	YKR066C	*IPT1*	YDR072C
CTT1	YGR088W	*KES1*	YPL145C
CYB2	YML054C	*LAC1*	YKL008C
CYB5	YNL111C	*LAG1*	YHL003C
DAN1	YJR150C	*LCB1*	YMR296C
DAP1	YPL170W	*LCB2*	YDR062W
DGA1	YOR245C	*LRO1*	YNR008W
DPL1	YDR294C	*MCR1*	YKL150W
ECM22	YLR228C	*MCT1*	YOR221C
ELO1	YJL196C	*MVD1*	YNR043W
ERG1	YGR175C	*NCP1*	YHR042W
ERG10	YPL028W	*NSG1*	YHR133C
ERG11	YHR007C	*OLE1*	YGL055W
ERG12	YMR208W	*OSH2*	YDL019C
ERG13	YML126C	*OSH3*	YHR073W
ERG2	YMR202W	*PET9*	YBL030C
ERG20	YJL167W	*PDR5*	YIL013C
ERG24	YNL280C	*PDR16*	YNL231C
ERG25	YGR060W	*POX1*	YGL205W
ERG26	YGL001C	*ROX1*	YPR065W
ERG27	YLR100W	*SCS7*	YMR272C
ERG28	YER044C	*SUT1*	YGL162W
ERG3	YLR056W	*TGL2*	YDR058C
ERG4	YGL012W	*TGL3*	YMR313C
ERG5	YMR015C	*TES1*	YJR019C
ERG6	YML008C	*TSC10*	YBR265W
ERG7	YHR072W	*TSC13*	YDL015C
ERG8	YMR220W	*UPC2*	YDR213W
ERG9	YHR190W	*YDC1*	YPL087W
FEN1	YCR034W	*YPC1*	YJR120W
		YSR3	YKR053C
