# Supplementary material for: Improvement of experimental testing and network training conditions with genome-wide microarrays for more accurate predictions of drug gene targets
Source: BMC Syst Biol. 2014 Jan 20;8:7. doi: 10.1186/1752-0509-8-7 (PMC3911882; doi:10.1186/1752-0509-8-7)
Supplement: Additional file 3 — (DNA_Gene_Set.pdf) - Orthogonal Gene Set: DNA replication and repair. [file 1752-0509-8-7-S3.pdf]

Gene Name	ORF Name	Gene Name	ORF Name
APN2	YBL019W	*POL32*	YJR043C
CDC9	YDL164C	*POL4*	YCR014C
DNA2	YHR164C	*PRI1*	YIR008C
DNL4	YOR005C	*PRI2*	YKL045W
DPB2	YPR175W	*RAD1*	YPL022W
DPB3	YBR278W	*RAD10*	YML095C
DPB4	YDR121W	*RAD14*	YMR201C
EXO1	YOR033C	*RAD2*	YGR258C
HOP2	YGL033W	*RAD26*	YJR035W
LIF1	YGL090W	*RAD27*	YKL113C
MAG1	YER142C	*RAD3*	YER171W
MEC1	YBR136W	*RAD4*	YER162C
MCM2	YBL023C	*RAD50*	YNL250W
MCM3	YEL032W	*RAD51*	YER095W
MCM4	YPR019W	*RAD52*	YML032C
MCM5	YLR274W	*RAD54*	YGL163C
MCM6	YGL201C	*RAD55*	YDR076W
MCM7	YBR202W	*RAD57*	YDR004W
MLH1	YMR167W	*RAD59*	YDL059C
MLH3	YPL164C	*RDH54*	YBR073W
MND1	YGL183C	*RFA1*	YAR007C
MRE11	YMR224C	*RFA2*	YNL312W
MSH2	YOL090W	*RFC1*	YOR217W
MSH3	YCR092C	*RFC2*	YJR068W
MSH6	YDR097C	*RFC3*	YNL290W
MUS81	YDR386W	*RFC4*	YOL094C
NEJ1	YLR265C	*RFC5*	YBR087W
NTG1	YAL015C	*RNH1*	YMR234W
NTG2	YOL043C	*RNH201*	YNL072W
OGG1	YML060W	*SGS1*	YMR190C
PMS1	YNL082W	*SRS2*	YJL092W
POL1	YNL102W	*TOP3*	YLR234W
POL12	YBL035C	*UNG1*	YML021C
POL2	YNL262W	*XRS2*	YDR369C
POL3	YDL102W	*YKU70*	YMR284W
POL30	YBR088C	*YKU80*	YMR106C
