# Supplementary material for: Improvement of experimental testing and network training conditions with genome-wide microarrays for more accurate predictions of drug gene targets
Source: BMC Syst Biol. 2014 Jan 20;8:7. doi: 10.1186/1752-0509-8-7 (PMC3911882; doi:10.1186/1752-0509-8-7)
Supplement: Additional file 4 — (Pyrimidine_Gene_Set.pdf) - Orthogonal Gene Set: Pyrimidine biosynthesis and metabolism. [file 1752-0509-8-7-S4.pdf]

**Gene Name**	**ORF Name**	**Gene Name**	**ORF Name**
ADK2	YER170W	*RPB3*	YIL021W
CDC21	YOR074C	*RPB4*	YJL140W
CDC8	YJR057W	*RPB5*	YBR154C
CDD1	YLR245C	*RPB7*	YDR404C
CPA1	YOR303W	*RPB8*	YOR224C
CPA2	YJR109C	*RPB9*	YGL070C
DAS2	YDR020C	*RPC10*	YHR143W-A
DCD1	YHR144C	*RPC11*	YDR045C
DFR1	YOR236W	*RPC19*	YNL113W
DUT1	YBR252W	*RPC25*	YKL144C
FCY1	YPR062W	*RPC31*	YNL151C
FUR1	YHR128W	*RPC34*	YNR003C
NPP1	YCR026C	*RPC37*	YKR025W
NPP2	YEL016C	*RPC40*	YPR110C
NTG1	YAL015C	*RPC53*	YDL150W
NTG2	YOL043C	*RPC82*	YPR190C
PHR1	YOR386W	*RPO21*	YDL140C
PNP1	YLR209C	*RPO26*	YPR187W
PPR1	YLR014C	*RPO31*	YOR116C
PUS2	YGL063W	*SDT1*	YGL224C
RET1	YOR207C	*TRR1*	YDR353W
RIM2	YBR192W	*TRR2*	YHR106W
RNR1	YER070W	*URA1*	YKL216W
RNR2	YJL026W	*URA10*	YMR271C
RNR3	YIL066C	*URA2*	YJL130C
RNR4	YGR180C	*URA3*	YEL021W
RPA12	YJR063W	*URA4*	YLR420W
RPA135	YPR010C	*URA5*	YML106W
RPA14	YDR156W	*URA6*	YKL024C
RPA190	YOR341W	*URA7*	YBL039C
RPA34	YJL148W	*URA8*	YJR103W
RPA43	YOR340C	*URH1*	YDR400W
RPA49	YNL248C	*URK1*	YNR012W
RPB10	YOR210W	*YND1*	YER005W
RPB11	YOL005C	*YNK1*	YKL067W
