# Supplementary material for: Improvement of experimental testing and network training conditions with genome-wide microarrays for more accurate predictions of drug gene targets
Source: BMC Syst Biol. 2014 Jan 20;8:7. doi: 10.1186/1752-0509-8-7 (PMC3911882; doi:10.1186/1752-0509-8-7)
Supplement: Additional file 5 — (RNA_Gene_Set.pdf) - Orthogonal Gene Set: RNA transport. [file 1752-0509-8-7-S5.pdf]

Gene Name	ORF Name	Gene Name	ORF Name
CDC33	YOL139C	*NUP49*	YGL172W
CRM1	YGR218W	*NUP84*	YDL116W
FUN12	YAL035W	*PAB1*	YER165W
GCD1	YOR260W	*POP1*	YNL221C
GCD11	YER025W	*POP3*	YNL282W
GCD2	YGR083C	*POP4*	YBR257W
GCD6	YDR211W	*POP5*	YAL033W
GCD7	YLR291C	*POP6*	YGR030C
GCN2	YDR283C	*POP7*	YBR167C
GCN3	YKR026C	*POP8*	YBL018C
GLE2	YER107C	*PRT1*	YOR361C
GSP1	YLR293C	*RNA1*	YMR235C
GSP2	YOR185C	*RPG1*	YBR079C
HCR1	YLR192C	*RPP1*	YHR062C
HSL7	YBR133C	*RPR2*	YIR015W
KAP95	YLR347C	*SEC13*	YLR208W
KRE28	YDR532C	*SEH1*	YGL100W
LOS1	YKL205W	*SMT3*	YDR510W
LSR1	LSR1	*SUI1*	YNL244C
MEX67	YPL169C	*SUI2*	YJR007W
MLP1	YKR095W	*SUI3*	YPL237W
MLP2	YIL149C	*TEF1*	YPR080W
MSN5	YDR335W	*TEF2*	YBR118W
MTR10	YOR160W	*TGS1*	YPL157W
MTR2	YKL186C	*THO2*	YNL139C
NAM7	YMR080C	*TIF1*	YKR059W
NDC1	YML031W	*TIF11*	YMR260C
NIC96	YFR002W	*TIF2*	YJL138C
NIP1	YMR309C	*TIF3*	YPR163C
NMD2	YHR077C	*TIF34*	YMR146C
NMD3	YHR170W	*TIF35*	YDR429C
NUP116	YMR047C	*Tif4631p*	YGR162W
NUP133	YKR082W	*Tif4632p*	YGL049C
NUP170	YBL079W	*TIF5*	YPR041W
NUP188	YML103C	*TRZ1*	YKR079C
NUP192	YJL039C	*UBC9*	YDL064W
		YRB1	YDR002W
