# Supplementary material for: Improvement of experimental testing and network training conditions with genome-wide microarrays for more accurate predictions of drug gene targets
Source: BMC Syst Biol. 2014 Jan 20;8:7. doi: 10.1186/1752-0509-8-7 (PMC3911882; doi:10.1186/1752-0509-8-7)
Supplement: Additional file 6 — (Glyc_Pent_Gene_Set.pdf) - Orthogonal Gene Set: Glycolysis and pentose phosphate. [file 1752-0509-8-7-S6.pdf]

Gene Name	ORF Name	Gene Name	ORF Name
ACS2	YLR153C	*NQM1*	YGR043C
ADH1	YOL086C	*PCK1*	YKR097W
ADH2	YMR303C	*PDA1*	YER178W
ADH3	YMR083W	*PDB1*	YBR221C
ADH4	YGL256W	*PDC1*	YLR044C
ADH5	YBR145W	*PDC5*	YLR134W
ADH6	YMR318C	*PDC6*	YGR087C
ADH7	YCR105W	*PFK1*	YGR240C
ALD2	YMR170C	*PFK2*	YMR205C
ALD3	YMR169C	*PGI1*	YBR196C
ALD4	YOR374W	*PGK1*	YCR012W
ALD5	YER073W	*PGM1*	YKL127W
ALD6	YPL061W	*PGM2*	YMR105C
CDC19	YAL038W	*PGM3*	YMR278W
ENO1	YGR254W	*PRS1*	YKL181W
ENO2	YHR174W	*PRS2*	YER099C
ERR1	YOR393W	*PRS3*	YHL011C
ERR3	YMR323W	*PRS4*	YBL068W
FBA1	YKL060C	*PRS5*	YOL061W
FBP1	YLR377C	*PYK2*	YOR347C
GAL10	YBR019C	*RBK1*	YCR036W
GLK1	YCL040W	*RKI1*	YOR095C
GND1	YHR183W	*RPE1*	YJL121C
GND2	YGR256W	*SFA1*	YDL168W
GPM1	YKL152C	*SOL1*	YNR034W
GPM2	YDL021W	*SOL2*	YCR073W-A
GPM3	YOL056W	*SOL3*	YHR163W
HXK1	YFR053C	*SOL4*	YGR248W
HXK2	YGL253W	*TAL1*	YLR354C
hypothetical protein	YKR043C	*TDH1*	YJL052W
hypothetical protein	YMR099C	*TDH2*	YJR009C
hypothetical protein	YDR248C	*TDH3*	YGR192C
hypothetical protein	YOR283W	*THI3*	YDL080C
IRC15	YPL017C	*TKL1*	YPR074C
LAT1	YNL071W	*TKL2*	YBR117C
LPD1	YFL018C	*TPI1*	YDR050C
		ZWF1	YNL241C
