# Supplementary material for: Improvement of experimental testing and network training conditions with genome-wide microarrays for more accurate predictions of drug gene targets
Source: BMC Syst Biol. 2014 Jan 20;8:7. doi: 10.1186/1752-0509-8-7 (PMC3911882; doi:10.1186/1752-0509-8-7)
Supplement: Additional file 7 — (Mitosis.pdf) - Orthogonal Gene Set: Mitosis. [file 1752-0509-8-7-S7.pdf]

Gene Name	ORF Name	Gene Name	ORF Name
AME1	YBR211C	*IRR1*	YIL026C
ASK1	YKL052C	*LTE1*	YAL024C
BFA1	YJR053W	*MAD1*	YGL086W
BIM1	YER016W	*MAD2*	YJL030W
BRN1	YBL097W	*MAD3*	YJL013C
BUB1	YGR188C	*MCD1*	YDL003W
BUB2	YMR055C	*MET30*	YIL046W
BUB3	YOR026W	*MIH1*	YMR036C
CAK1	YFL029C	*MOB1*	YIL106W
CDC14	YFR028C	*MPS1*	YDL028C
CDC15	YAR019C	*MTW1*	YAL034W-A
CDC5	YMR001C	*NET1*	YJL076W
CDC55	YGL190C	*NNF1*	YJR112W
CDH1	YGL003C	*NSL1*	YPL233W
CEP3	YMR168C	*OKP1*	YGR179C
CHK1	YBR274W	*PDS1*	YDR113C
CKS1	YBR135W	*PDS5*	YMR076C
CLB1	YGR108W	*PPH21*	YDL134C
CLB2	YPR119W	*PPH22*	YDL188C
CTF19	YPL018W	*SCC2*	YDR180W
DAM1	YGR113W	*SCC4*	YER147C
DBF2	YGR092W	*SGO1*	YOR073W
DBF20	YPR111W	*SLK19*	YOR195W
DMA1	YHR115C	*SMC1*	YFL008W
DMA2	YNL116W	*SMC2*	YFR031C
ECO1	YFR027W	*SMC3*	YJL074C
ESP1	YGR098C	*SMC4*	YLR086W
FIN1	YDR130C	*SPC105*	YGL093W
FOB1	YDR110W	*SPO12*	YHR152W
GAC1	YOR178C	*SWE1*	YJL187C
GIN4	YDR507C	*SWI5*	YDR146C
HHT1	YBR010W	*TEM1*	YML064C
HHT2	YNL031C	*TID3*	YIL144W
HSL1	YKL101W	*TPD3*	YAL016W
HSL7	YBR133C	*ULP2*	YIL031W
IBD2	YNL164C	*YCG1*	YDR325W
IPL1	YPL209C	*YCS4*	YLR272C
